# Dissecting the Roles of LncRNAs in the Development of Periventricular White Matter Damage

**DOI:** 10.3389/fgene.2021.641526

**Published:** 2021-04-30

**Authors:** Xinyu Wang, Heng Liu, Xiaoli Liao, Lixing Qiao, Lihua Zhu, Shun Wu, Yan Zhou, Yi Zhang, Bangbang Li, Lili Lin, Jingjing Ma, Qianying Gu, Jiaping Shu

**Affiliations:** ^1^Department of Pediatrics, Zhongda Hospital, Southeast University, Nanjing, China; ^2^Institute of Clinical, Jiangsu Health Vocational College, Nanjing, China

**Keywords:** long non-coding RNA, premature infants, periventricular white matter damage, human blood, RNA sequencing

## Abstract

Long non-coding RNA (LncRNA) has high expression in the brain. Animal studies have shown that lncRNA plays an important role in brain functions and mediates the development of many neurological diseases. However, data on the expression of lncRNAs and the clinical significance in prematurely born infants with diseases such as periventricular white matter damage (PWMD) remains scant. Here, we compared the expression of the lncRNAs in whole blood samples obtained from prematurely born infants with PWMD with samples from prematurely born infants without PWMD. Our data demonstrated differential expression of the lncRNAs between the two groups. Further, we showed that the lncRNAs play important roles in the development of PWMD. Our findings give insights into the functions of the lncRNAs in PWMD and provide evidence for the improvement of diagnostic and treatment strategies in infants with PWMD.

## Introduction

Periventricular white matter damage (PWMD) is a brain damage condition that contributes to premature births. Besides, PWMD leads to neurological diseases, including convulsions, cerebral palsy, epilepsy, or cognitive/motor dysfunction, as well as affecting the quality of life in children (Lee, 2017; Liu et al., 2019). The development of PWMD has been associated with fetal intrauterine hypoplasia and extrauterine growth environment. However, data on the molecular mechanisms that define the occurrence of PWMD remains scant. The data would help inform diagnosis and treatment strategies for PWMD.

Premature infants with PWMD might not have typical clinical disease manifestation and their organs or nervous system is relatively immature at birth. Brain imaging tools such as cranial ultrasound and magnetic resonance imaging (MRI) are the mainstay methods in the diagnosis of PWMD. However, the imaging methods are limited by hysteresis. The lack of understanding of the PWMD dynamics greatly hinders early diagnosis, proper interventions, and evaluation of the disease prognosis. Thus, an understanding of the molecular mechanisms of PWMD will not only optimize therapeutic strategies but improve the overall outcomes of PWMD.

With the development of molecular biology tools, studies have demonstrated the roles of long non-coding RNAs (lncRNAs) at multiple levels, including transcriptional, post-transcriptional, as well as epigenetic levels (Qureshi and Mehler, 2013). The high expression of the lncRNAs in the brain tissues does not only mediate the development and differentiation of nerve cells, but also plays an important role in the development and recovery of brain damage (Chen et al., 2019). On the other hand, some studies have shown that the lncRNAs modulate the pathophysiology of neurological and mental diseases (Lipovich et al., 2014; Andersen and Lim, 2018).

Besides, previous studies have demonstrated differential expression of the lncRNAs in the brain tissues from hypoxic-ischemic brain damage (HIBD) in rats, as well as the lncRNAs’ participation in brain damage by regulating mRNA expression (Lee et al., 2015; Zhao et al., 2015; Jablonska et al., 2016). However, there are no data on the potential shifts in lncRNA expression in patients with PWMD. Here, we evaluated the differential expression of the lncRNAs in peripheral venous blood from premature infants. We focused on assessing the differentially expressed (DE) lncRNAs and mRNAs, as well as their functional correlation. Our data robustly demonstrate differential lncRNA expression between the PWMD group and the control group. We then selected the DE lncRNAs and constructed a lncRNA-mRNA co-expression network. In addition, we conducted *in silico* analyses, such as Gene Ontology (GO) analysis, Kyoto Encyclopedia of Genes and Genomes (KEGG) pathway, and Gene Set Enrichment Analysis (GSEA), on the lncRNAs, to predict the underlying functions in PWMD. Together, we provide new insights into the mechanisms of PWMD which could inform the development of diagnostic and prognostic tools for PWMD.

## Materials and Methods

### Ethics Statements

Our study protocols were approved by the Clinical Research Ethics Committee of Zhongda Hospital Affiliated to Southeast University (2018ZDKYSB119) prior to the commencement of the study. We obtained written informed consent from the parents of all the children who participated in the study.

### Samples

We collected the samples following the protocol described in our previous study (Qiao et al., 2020). Thereafter, a total of six samples (N12, N15, N27 from PWMD group and N13, N55, N78 from control group) were used for RNA sequencing (Table 1).

**TABLE 1:** Sample grouping.

**Number**	**Sample**	**Group**	**Gestational (Weeks)**	**Birth weight (g)**
1	N12	PWMD	27 + 1	1170
2	N15	PWMD	26 + 5	990
3	N27	PWMD	27 + 2	1100
4	N13	Control	29 + 1	1230
5	N55	Control	29 + 1	1200
6	N78	Control	27 + 3	800

### RNA Sequencing, Read Mapping, and Differential Expression Analysis

RNA sequencing was performed by Aksomics, Shanghai, China. Out of the six samples submitted for inspection, a total of 1–2 μg RNA per sample was extracted for sequence library construction. The quality of the constructed library was evaluated by Agilent 2100 Bioanalyzer, and then the library was quantified using qPCR. The mixed libraries were sequenced using Illumina HiSeq 4000 sequencer. RNA-seq library was prepared using KAPA Stranded RNA-Seq Library Prep Kit (Illumina), which incorporates dUTP into the second cDNA strand, rendering specificity to the RNA-seq library strand.

Image analysis and base calling were performed using Solexa pipeline v1.8 (Off-Line Base Callersoftware, v1.8). We then assessed the quality of the sequences using the FastQC software. The trimmed reads (trimmed 5′, 3′adaptor bases using cutadapt) were aligned to the reference genome using Hisat2 software. Genome version used in reads mapping is GenCode GRCh37. On the other hand, we estimated the transcript abundance in each sample using StringTie (version 1.3.3b), while the FPKM (Fragments Per Kilobase of gene/transcript per Million mapped fragments) value for the gene or transcript was calculated by the R package Ballgown, and then represented by the average log2(FPKM+1). In addition, we evaluated the differentially expressed genes (DEGs) and transcripts using the R package Ballgown. The novel genes and transcripts were predicted from assembled results by comparing to the annotated reference using StringTie and Ballgown, while the coding potential for the sequences was assessed by CPAT. The genes or transcripts that significantly differed between the groups were defined by a 1.5-fold change, with a *p*-value < 0.05.

### RNA Isolation and Quality Control

Within 24 h postpartum, 0.5 ml venous blood samples were drawn from the infants into EDTA tubes and were preserved in liquid nitrogen. The blood samples were lysed by TRIzol LS Reagent (Thermo Fisher Scientific, United States), and then the total RNA was extracted with chloroform. Thereafter, the samples were precipitated with absolute ethanol and rinsed, followed by purification and recovery using the RNAprep pure Cell/Bacteria Kit (Tiangen Biotech, China), following the manufacturer’s instructions. RNA completeness and purity were monitored by resolving on a 1% agarose gels. The concentration and purity of the RNA was assessed using a NanoDrop spectrophotometer ND-1000. Samples observed at optical density (OD) _260/280_ with a ratio between 1.8 and 2.1 or OD_260/230_ with a ratio > 1.8 were considered eligible.

### Quantitative Real-Time PCR (qRT-PCR) Assay

The total RNA extracted from the blood samples was used to synthesize cDNA by HiScriptII Q RT SuperMix for qPCR (+g DNA wiper) kit and the quantitative real-time PCR (qRT-PCR) experiments were performed with the ChamQ^TM^ Universal SYBR qPCR Master Mix kit (Vazyme Biotech, Nanjing, China). Briefly, we conducted the amplification in a total reaction volume of 10 ul. The reaction included 5 ul SYBR qPCR Master Mix, 0.5 ul forward primer, 0.5 ul reverse primer, 1 ul cDNA and 3 ul H_2_O. The PCR amplification protocol involved 5 min denaturation at 95°C, followed by 40 repetitive cycles (10 s at 95°C and 30 s at 60°C). The relative expression of the genes was calculated by ΔCT method and all the samples were normalized to GAPDH. The reactions were conducted in triplicates. The primers used in this experiment are as shown in Table 2.

**TABLE 2:** Primers for qRT-PCR.

**LncRNA**	**Forward primer (5′→3′)**	**Reverse primer (5′→3′)**
FCGR2A-212	AGGCAGGAGTCCAGAAGTAG	AGAAGTCATTCCAGGCAGAT
FAM157C-202	CTGGCATTTCACAATCAACA	GATGGGATTTGGGAATAGGT
LINC01270-202	TGAAAGGTGGCGGGAAGTGGG	AGGCTGGCCTGGGGTTGAATG
BID-206	TCATCGTAGCCCTCCCACT	ACAACGGTTCCAGCCTCA
YBX3-210	CTGAGGAGCCTGGTGTTA	GTCTGTTCGCCGTGGATA
GAS5-209	ATTCTGAGTCCTTTGCTTCTT	TAATGGTTCTGCTCCTGGTAA
GAPDH	CGCTGAGTACGTCGTGGAGTC	GCTGATGATCTTGAGGCTGTTGTC

### Functional Enrichment Analysis

To infer the possible roles of the regulatory non-coding RNAs, we conducted functional enrichment analysis of the target genes. GO analysis was performed via^1^ to find out which specific feature terms are most associated with DEmRNAs. Besides, we used KEGG database^2^ to assess the DEGs, as well as the most relevant functional terms. *P*-value indicates the significance of the correlation in both the GO and KEGG analysis. A smaller *p*-value indicates that the genes are more likely to have a descriptive function corresponding to the terms.

In addition, we used the GSEAPrerank in GSEA software to perform the conduct enrichment analyses (GO and KEGG pathway analyses) of the mRNA related to the lncRNA through its pairwise correlation. We then extracted the first five items as sorted by FDR *q*-values in all lncRNAs related with the enrichment analysis results were extracted respectively, and the lncRNA-Term correlation matrix was constructed with the Normalized Enrichment Score (NES), corresponding to each entry. In addition, the heat maps of the GO and KEGG pathway entries were also created respectively.

### Co-expression Analysis and Network Construction

Co-expression analysis is often used to explore the correlation between coding and non-coding RNAs, thus forecasting the function of the non-coding RNA. All DE lncRNAs and DE mRNAs between the two groups were selected for the co-expression analysis. We calculated the Pearson correlation coefficient (PCC) of each of the lncRNAs and mRNAs. An absolute value of PCC > 0.95 and a *p*-value < 0.05 were used to define strong co-expression correlation between two genes. A PCC > 0 indicated positive correlation whereas a PCC < 0 showed that the two are negatively correlated. We then used the co-expression data to construct a positive or negative co-expression network. For the purposes of clarity and conciseness, we only selected the top 10 up-regulated or down-regulated lncRNAs for visualization using Cytoscape v3.7.2.

### Statistical Analyses

We performed statistical analyses using SPSS software 21.0 (SPSS Inc., Chicago, IL, United States). Student’s *t*-test was also used to assess the differences between groups. And *p-*value < 0.05 was considered statistically significant.

## Results

### LncRNAs Profile Among the Groups

**FIGURE 1:**
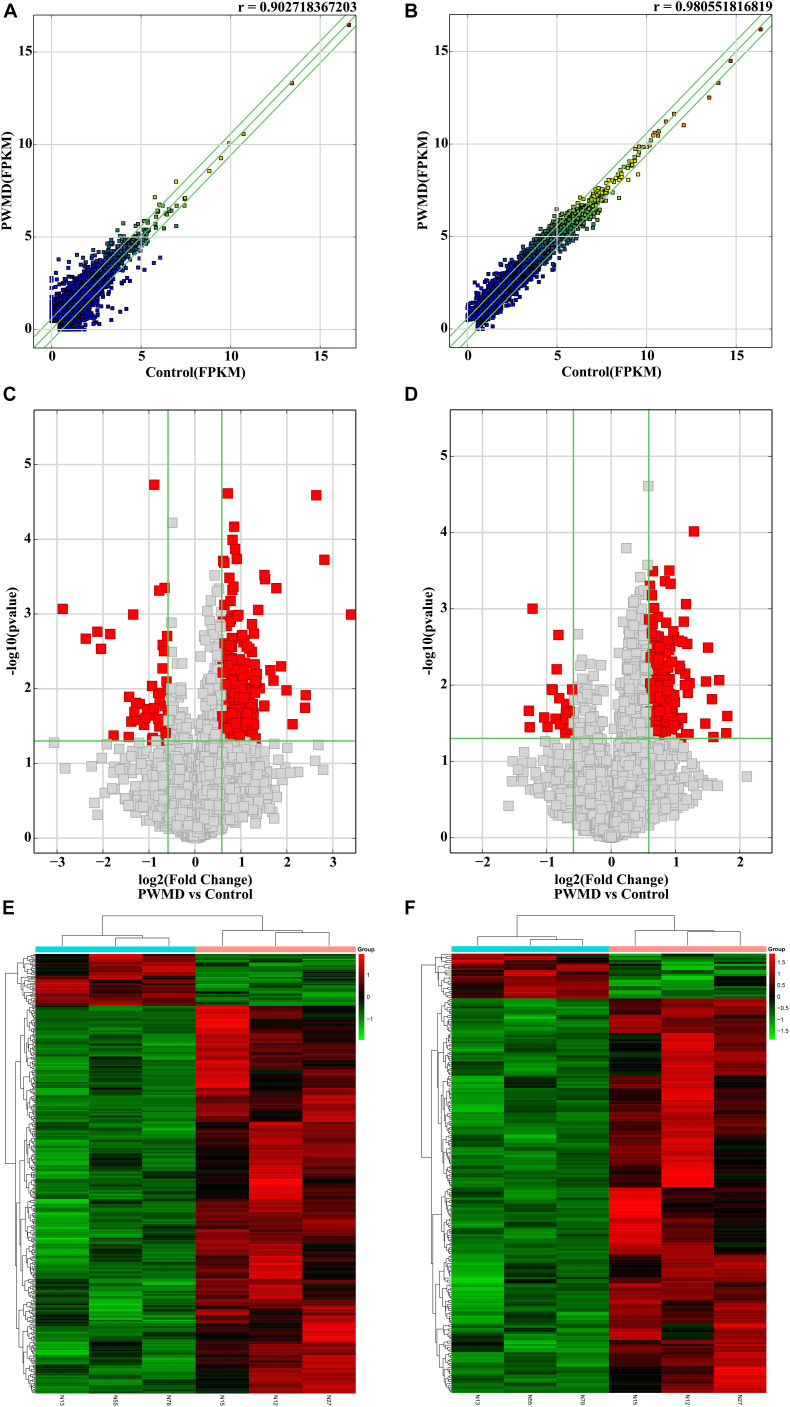
The RNA sequencing data from the lncRNAs and mRNAs obtained from the PWMD and Control groups. Scatter plots of the FPKM of **(A)** lncRNAs and **(B)** mRNAs, the *r* value represents the correlation between the groups. Volcano plots represent differentially expressed **(C)** lncRNAs and **(D)** mRNAs, red square dots represent differential expression, gray square dots represent lack of differential expression, the upper area of the green horizontal line represents *p*-value < 0.05, the left region of the left green vertical line and the right region of the right green vertical line represent the absolute value of the fold change > 1.5. The heatmap shows the clusters of **(E)** lncRNAs and **(F)** mRNAs differentially expressed in the PWMD group. N13, N55, N78 represent the group Control; N12, N15, N27 represent the PWMD group. Red patches represent higher relative expression, while green patches represent lower relative expression. *P*-value < 0.05 was considered statistically significant.

Out of the total 11156 lncRNAs detected, 362 (3.24%) were DE in the PWMD group (fold change > 1.5, *p*-value < 0.05), where 321 were up-regulated while the remaining 41 were down-regulated. The top 10 up- and down-regulated lncRNAs are shown in Table 3. The differences between the two groups of samples were visualized by scatter plots, volcano plots, and hierarchical clustering heat maps (Figure 1). We also analyzed the genome annotation of the lncRNAs (Supplementary Tables 1,2).

**TABLE 3:** Top 10 up-regulated and down-regulated lncRNAs in the PWMD blood samples compared with control blood samples.

**Trans_Name**	**Locus**	**Fold_Change**	***p*_value**
**Up-regulation**			
FCGR2A-212	chr1:161487765-161492889	10.52652485	0.001017468
ILF3-205	chr19:10798281-10803074	7.043760415	0.00018809
IP6K1-206	chr3:49822913-49823627	6.235289045	2.57779*E*-05
SLC6A6-206	chr3:14444148-14499534	5.317461563	0.012220975
PRKACA-202	chr19:14202509-14219202	5.26366299	0.018005365
CSNK1G3-204	chr5:122847885-122881650	4.361087397	0.029867325
TFDP1-205	chr13:114239058-114288314	3.979541944	0.010520901
RPS10-204	chr6:34385231-34393561	3.665823203	0.005025761
ZMYM1-202	chr1:35544992-35581455	3.41259633	0.000451207
KCNE1-207	chr21:35881754-35884505	3.268384103	0.007789135
**Down-regulation**			
YBX3-210	chr12:10851916-10855074	0.136220343	0.000854955
GUCD1-212	chr22:24938798-24940051	0.192716136	0.002140712
CPNE1-223	chr20:34246852-34252822	0.22964537	0.001726334
NPEPPS-204	chr17:45600311-45669911	0.242748315	0.002928789
TMEM50A-205	chr1:25664811-25687756	0.293433584	0.04237207
GAS5-209	chr1:173833039-173838020	0.368369589	0.044256588
LINC02446-201	chr12:10705962-10710648	0.370793022	0.012845616
LINC01506-202	chr9:71158457-71161505	0.38332337	0.027564071
DDX11L9-201	chr15:102516761-102519296	0.401316234	0.020499495
BID-206	chr22:18220824-18257261	0.411436698	0.015491064

### Validation of Dysregulated LncRNAs in PWMD

**FIGURE 2:**
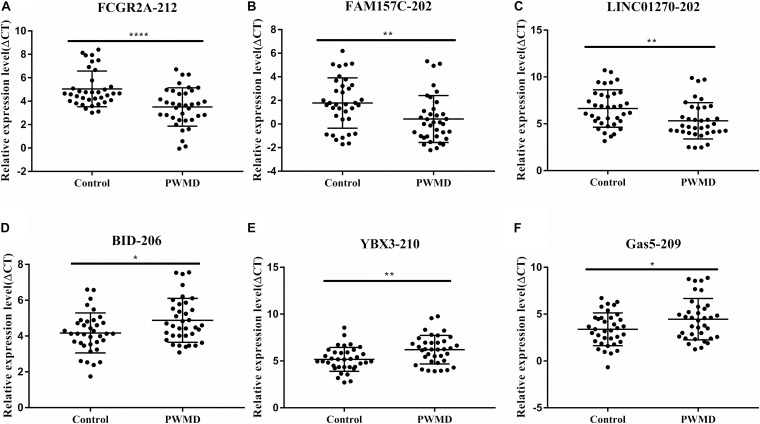
Validation of six differentially expressed LncRNAs **(A–F)** in 36 blood samples from the two groups using qRT-PCR. The ordinate is ΔCT value, ΔCT = CT (lncRNA)-CT (internal control), and the expression level of lncRNA is negatively associated with ΔCT value. Student’s *t*-test was used for comparisons between the two groups (*p*-value < 0.05). *P < 0.05; **P < 0.01; ****P < 0.0001.

To validate the sequencing data, we randomly selected six DE lncRNAs from 72 samples (36 control samples and 36 PWMD) for qRT-PCR verification (Table 4). Consistent with the lncRNAs expression data from RNA sequencing, qRT-PCR analysis showed an up-regulation of FCGR2A-212, FAM157C-202, and LINC01270-202, while BID-206, YBX3-210, and GAS5-209 were down-regulated in the PWMD group (Figure 2). These data further confirmed the differential expression of these six lncRNAs in the peripheral venous blood of patients with PWMD. Meanwhile, we have reason to consider that the accuracy of RNA-sequencing results is credible.

**TABLE 4:** Baseline physiological variables.

**Newborn characteristics**	**Control group**	**PWMD group**	***p*-value**
Sex (Male/Female)	16/20	19/17	n.s
Gestational (Weeks)	32.57 ± 2.28	30.28 ± 2.1	<0.05
Weight (g)	1870 ± 220	1460 ± 310	<0.05
Delivery mode (eutocia/cesarean)	25/16	24/17	n.s

### Functional Characterization of the DE LncRNAs in the PWMD Group

To dissect the possible functions of DE lncRNAs, we conducted GO enrichment analysis of the mRNAs. GO analysis consists of three aspects: biological processes (BP), molecular functions (MF), and cellular components (CC).

**FIGURE 3:**
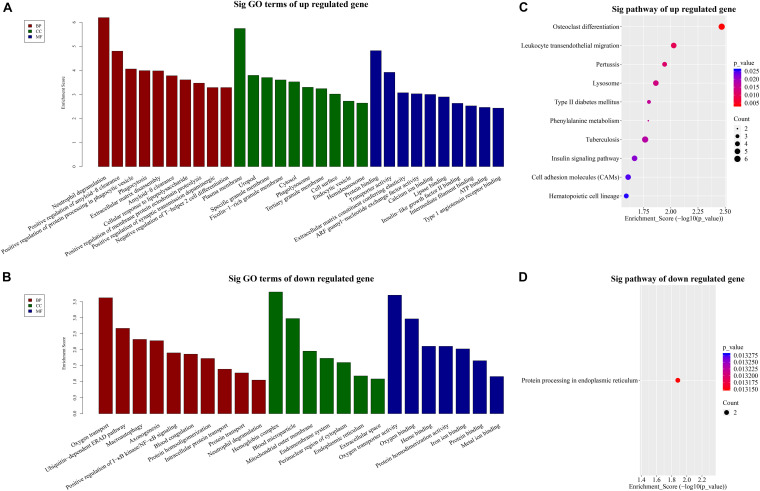
GO and KEGG pathway analysis. TOP 10 GO terms enriched in BP, CC, or MF among the **(A)** up-regulated mRNAs and **(B)** down-regulated mRNAs. Significantly enriched KEGG pathways in **(C)** up-regulated mRNAs and **(D)** down-regulated mRNAs.

Our BP terms showed that the up-regulated mRNAs are mainly enriched in neutrophil degranulation, positive regulation of amyloid-β clearance, cellular response to lipopolysaccharide, or positive regulation of synaptic transmission dopaminergic, etc. (Figure 3A). On the other hand, down-regulated mRNAs were mapped to oxygen transport, ubiquitin dependent ERAD pathway, axon-genesis, positive regulation of I-κB kinase/NF-κB signaling, etc. (Figure 3B).

Similarly, the MF analysis showed that the up-regulated mRNAs were mainly enriched in these GO terms: protein binding, transporter activity, extra-cellular matrix constituent conferring elasticity or ARF guanyl-nucleotide exchange factor activity, etc. (Figure 3A). The suppressed mRNAs were mainly involved in oxygen transporter activity, oxygen binding, heme binding, or iron binding (Figure 3B).

In addition, the CC data demonstrated that the up-regulated mRNAs were enriched in plasma membrane, uropod or ficolin-1-rich granule membrane, etc. (Figure 3A). The down-regulated mRNAs were shown to be concentrated in hemoglobin complex, blood microparticle, mitochondrial outer membrane, or endomembrane system, etc. (Figure 3B).

Furthermore, to provide insights into the roles of the DE lncRNAs in signaling pathways, we conducted KEGG pathway analysis of DE mRNAs. Our results indicated that 29 pathways corresponded to the up-regulated mRNAs while one pathway was mediated by the down-regulated mRNAs. Besides, osteoclast differentiation emerged to be the most enriched pathway in the up-regulated protein-coding genes (Figure 3C). Protein processing in endoplasmic reticulum was the only pathway affected by the down-regulated genes (Figure 3D).

**FIGURE 4:**
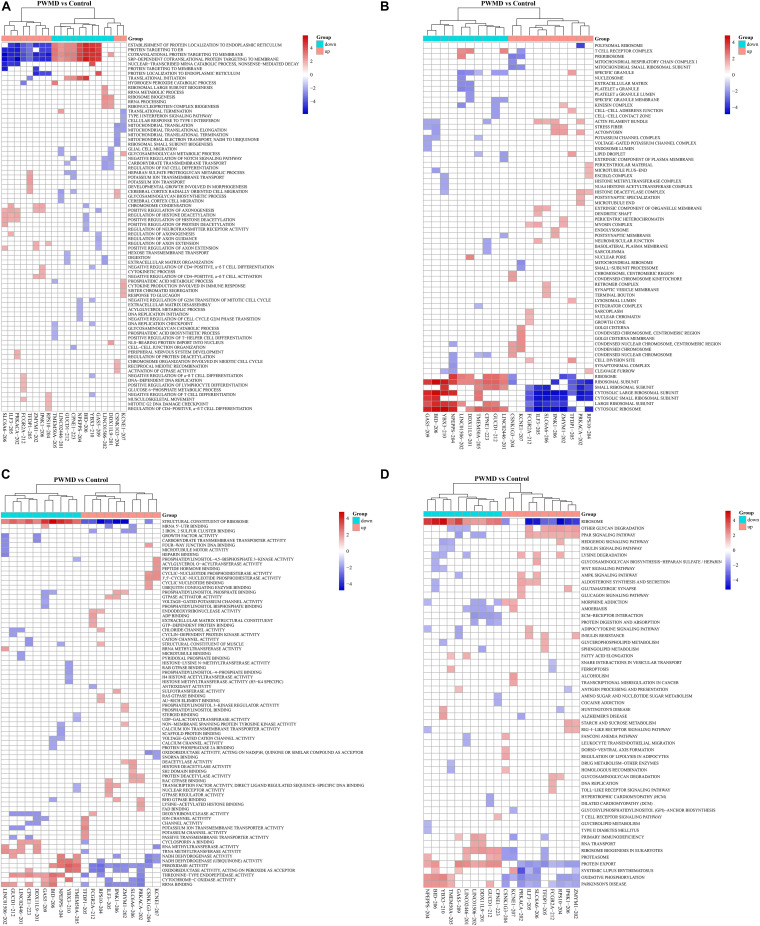
The cluster heat map for enrichment of **(A)** BP, **(B)** CC, **(C)** MF, and **(D)** KEGG among the differentially expressed lncRNAs gene sets. Each row represents a functional entry while each column represents an lncRNA.

We then employed GSEAPreranked in GSEA software to perform GSEA on the DE lncRNAs. The results have been presented by heat maps (Figure 4).

### Co-expression Analysis of the DE LncRNAs and DE mRNAs in the PWMD Group

**FIGURE 5:**
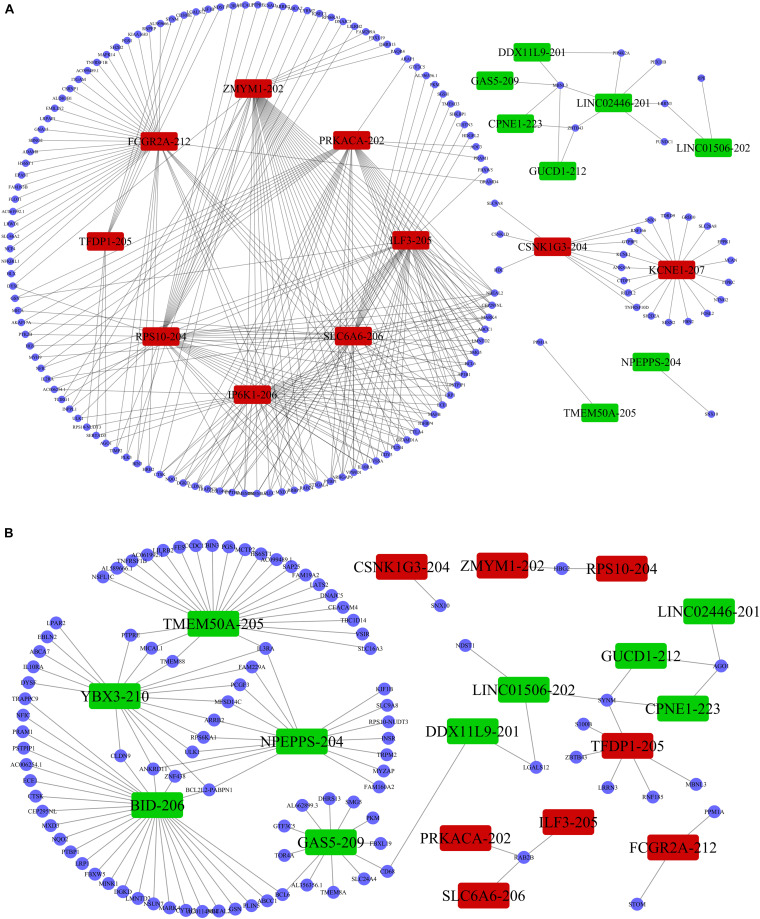
LncRNA-mRNA co-expression analysis. The **(A)** positive co-expression network and the **(B)** negative co-expression networks were conducted individually. Rectangular nodes represent DE lncRNAs, red color shows up-regulation while the green color denotes down-regulation of the expression. Blue circular nodes represent DE mRNAs.

Previous studies demonstrated that lncRNAs regulate the expression of target-genes locally (in *cis*) or non-locally (in *trans*) (Mowel et al., 2017; Kopp and Mendell, 2018). Besides, the functions of most of the lncRNAs remain undetermined and possess challenges in direct detection. However, the functions can be reflected in their associated mRNAs. Accordingly, we performed co-expression analysis between the DE lncRNAs and DE mRNAs to help us understand the roles of the DE lncRNAs in the PWMD group. Our analysis showed a total of 5691 positively correlated lncRNA-mRNA pairs and 801 negatively corelated lncRNA-mRNA pairs. The correlation was obtained from the PCC data involving 362 DE lncRNAs and 216 DE mRNAs. Thereafter, based on the co-expression patterns, we constructed corresponding coding non-coding gene co-expression (CNC) networks (Figure 5). The positive co-expression network contained 343 unique lncRNAs and 207 unique mRNAs. LINC01002-214, MEG8-201, CCDC88B-204, LINC02256-201, and LINC01270-201. lncRNAs had the highest degree of association. On the other hand, the negative co-expression network contained 801 lncRNA-mRNA pairs, involving 214 unique lncRNAs and 146 unique mRNAs. BID-206, TMEM50A-205, SERTAD3-205, YBX3-210, and NPEPPS-204 lncRNAs had the strongest association.

## Discussion

There is increased attention toward studies on the functions of lncRNAs (Rinn et al., 2007; Cech and Steitz, 2014; Engreitz et al., 2016). Previous studies have shown that lncRNAs display dynamic expression patterns in the human brain with a high degree of spatial and temporal specificity. Aberrant lncRNA expression may not only cause brain dysfunction but mediate tumorigenesis and neuropsychiatric disorders (Briggs et al., 2015). Although there are data showing variations in lncRNA expression in animal models of PWMD (Wang et al., 2020), little is known about lncRNAs shifts in humans with PWMD. Because of the technical challenges in obtaining brain tissues from infants, we adopted liquid biopsy to analyze peripheral blood and attempt to understand the molecular mechanisms of the disease (Pantel and Alix-Panabieres, 2014). We have previously carried out a preliminary study on circRNA expression signatures in peripheral venous blood from PWMD patients and performed functional analysis (Qiao et al., 2020). Here, we interrogated the expression profile of lncRNAs in infants with PWMD.

Our differential expression analysis showed 362 dysregulated lncRNAs (fold change > 1.5, *p*-value < 0.05) in the PWMD group. Interestingly, most of the lncRNAs were up-regulated (88.67%), which might indicate PWMD. During necroptotic, there is export of intracellular contents, such as hepatic enzymes, into body fluids, which are associated with liver cell damage (Gui et al., 2015). We hypothesized that a similar phenomenon might exist in the lncRNA in neural cells. There are, however, limited data on how the lncRNAs break through the blood–brain barrier (BBB). It is therefore feasible that exosomes, organelles that contain non-coding RNAs, might serve as vehicles for the lncRNAs across the BBB into systemic circulation (Ramachandran and Palanisamy, 2012). Another study showed that PWMD leads to hyperpermeability of the BBB as a result of the activation of glial cells (Abbott et al., 2006), thus facilitating the movement of lncRNAs across the BBB. Moreover, since the neonatal BBB is immature and more permeable (Goines et al., 2011), the lncRNAs might be directly associated with PWMD. However, this hypothesis needs further interrogation.

In addition, previous studies have shown that lncRNAs act on the coding genome via *cis*- or *trans*-regulation (Chalei et al., 2014). Thus, lncRNAs and mRNAs might have a co-expression linkage. Here, we adopted the PCC to assess the strength of association between the two variables. The data showed that LINC01002-214 had the highest negative correlation with mRNA PPM1A. Whereas the functions of the LINC01002-214 have not been defined, its negative correlation with mRNA PPM1A mediates a significant role in cellular functions such as cell cycle progression, apoptosis, and differentiation (Chuderland et al., 2012). Besides, some studies have reported that over-expression of PPM1A in nerve cells results in cell cycle arrest and apoptosis (Shohat et al., 2012), while the lack of PPM1A led to high expression of transforming growth factor-β related genes and subsequent promotion of angiogenesis (Dvashi et al., 2014). On the other hand, oligodendrocyte precursor cells (OPCs) represent the most critical cell population involved in white matter recovery after damage, arresting of cell cycle which leads to inhibited proliferation (Sun et al., 2016) and angiogenesis, thus promoting germination of the OPCs (Miyamoto et al., 2014). It is important to note that, whereas LINC01002-214 was up-regulated, PPM1A was down-regulated in the PWMD group. This finding strongly suggests that LINC01002-214 might be conferring a protective effect by suppressing the PPM1A in response to brain damage.

**FIGURE 6:**
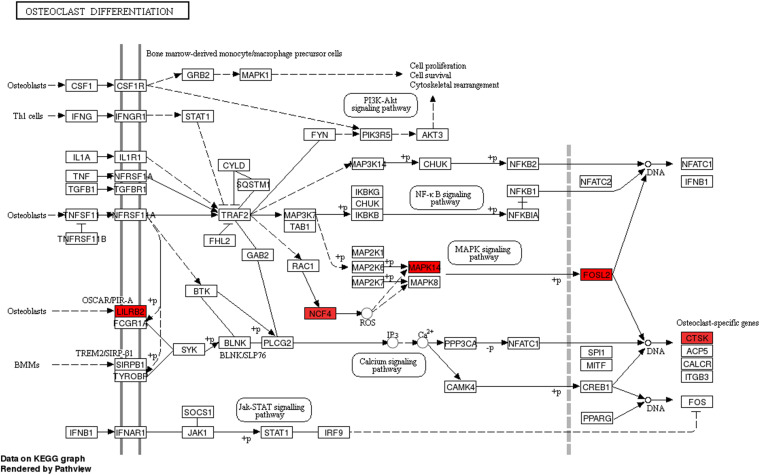
Pathway analysis of the Osteoclast Differentiation signaling pathway. Red rectangles represent mRNAs that are significantly differently expressed in this sequencing.

We then assessed the degree of correlation of the lncRNAs. A lncRNA with more associated mRNAs raises the likelihood of playing a critical node in disease development. Interestingly, LINC01002-214 is also the most highly correlated lncRNA with 43 target genes. On the contrary, BID-206 is a down-regulated lncRNA with the strongest degree of negative correlation in the network. CTSK gene, one of the BID-206 target genes was shown to be associated with the osteoclast differentiation pathway. Conversely, PWMD functions independently of the osteoclast differentiation. Our data showed that Calcium and MAPK pathway, classic signaling pathways, were hidden in the osteoclast differentiation pathway (Figure 6). The activation of calcium pathway has been reported to confer protection against ischemic brain damage (Zundorf and Reiser, 2011), and calcium antagonists are widely used as neuroprotective drugs (Zhang et al., 2019). On the other hand, MAPK pathway plays an important role in HIBD (Hee et al., 2002). Surprisingly, CTSK is a conjunct downstream molecule that develops as a result of crosstalk between the two pathways (Figure 6). Thus, the data demonstrates that the inhibition of BID-206 expression would up-regulate CTSK, thus affecting the prognosis of immature white matter damage. Furthermore, in the negative correlation network, lncRNAs CNIH4-208, NPEPPS-204, and YBX3-210 have several overlapping target genes with BID-206, so we speculate that the potential function of these lncRNAs are similar to BID-206.

Besides the PCC and the degree of correlation, we also assayed the fold change in the DE lncRNAs in the PWMD group. Our data showed that YBX3-210 was the most suppressed lncRNA and its possible role has been analyzed above. So, we shift focus to the FCGR2A-212 which had the highest fold change in the up-regulated lncRNAs. The FCGR2A-212 target mRNAs are enriched in granulocyte threshing, response to lipopolysaccharide, angiogenesis, signal transduction, apoptosis, cell morphogenesis, and peptide serine phosphorylation. Besides, the target mRNAs participate in the vascular endothelial growth factor receptor signaling pathway, FoxO signaling pathway, and have a negative regulatory effect on Wnt signaling pathway. These factors are all implicated in the pathophysiological mechanisms of white matter damage (Cerisola et al., 2019). Thus, we alluded to the fact that the FCGR2A-212 might play a key player in the development of PWMD.

However, in traditional gene enrichment analysis, we treated only significant DEGs. This approach makes it easy to omit some insignificant DEGs which have important biological functions (Subramanian et al., 2005). Our GSEA data showed that peroxisome proliferator-activated receptor (PPAR) pathway was positively enriched in the up-regulated lncRNAs, which was not found in previous gene enrichment analysis. Nevertheless, it has been shown that activation of the PPAR pathway prevents inflammatory responses throughout the body and reverses tissue damage (Kurtak, 2014). Besides, PPAR pathway is an important factor in reparative processes of various forms of brain injury (Li et al., 2019; Oyagbemi et al., 2020; Tang et al., 2020). On the other hand, our analysis showed that Parkinson’s disease pathway was negatively enriched in the up-regulated lncRNAs. All the evidence above strongly suggests that these up-regulated lncRNAs may play a protective role in brain damage.

Additionally, there are some limitations in the present study. First, the sample size for RNA-seq may be too small. Moreover, we directly extracted RNA from peripheral blood in this study, but the total RNA was dominated by RNA from blood cells. In other words, the lncRNAs being studied were probably from various blood cells, which may not be linked directly to PWMD. This question has been evaluated at the beginning of the research, usually the content of RNA in serum is very low, so it is a more appropriate method to sequence RNA extracted from whole blood than from serum when the source of lncRNA in peripheral blood cannot be determined. Nonetheless, after enrichment analysis we did in fact find several lncRNAs which seem to be highly significantly related to PWMD. In the near future, we will detect the expression of these lncRNAs in serum and blood cells, respectively to reveal whether the candidate lncRNAs are mainly cell-free RNAs or mainly from blood cells.

In summary, our study was based on the perspective of human blood samples to explore the expression characteristics and functions of lncRNAs in PWMD. The data from the human sample is more convincing than that from the animal source due to the poor conservation of lncRNAs among different species (Hezroni et al., 2015). Compared with tissue, blood is easier to obtain and more non-invasive, so molecules derived from blood have more potential to become biomarkers. We found significant shifts in the expression profile of lncRNAs in peripheral blood from patients with PWMD and screened out some lncRNAs that may have great significance. These findings provide new insights into the development for finding diagnostic biomarkers or therapeutic targets for PWMD.

## Data Availability Statement

The datasets presented in this study can be found in online repositories. The names of the repository/repositories and accession number(s) can be found below: https://www.ncbi.nlm.nih.gov/genbank/, GSE131475.

## Ethics Statement

The studies involving human participants were reviewed and approved by the Clinical Research Ethics Committee of Zhongda Hospital Affiliated to Southeast University. Written informed consent to participate in this study was provided by the participants’ legal guardian/next of kin.

## Author Contributions

LQ: conceptualization, supervision, review and editing, and project administration. XW: formal analysis, data curation, and writing-original draft. HL: methodology, review and editing. LZ: funding acquisition. XL, YaZ, YiZ, BL, SW, LL, JM, and QG: resources. JS: polishing. All authors contributed to the article and approved the submitted version.

## Conflict of Interest

The authors declare that the research was conducted in the absence of any commercial or financial relationships that could be construed as a potential conflict of interest.

## Footnotes

1 https://david.ncifcrf.gov/

2 https://www.genome.jp/kegg/pathway.html
