# Supplementary material for: Dissecting the Roles of LncRNAs in the Development of Periventricular White Matter Damage
Source: Front Genet. 2021 Apr 30;12:641526. doi: 10.3389/fgene.2021.641526 (PMC8120246; doi:10.3389/fgene.2021.641526)
Supplement: Supplementary file 1 [file Data_Sheet_1.PDF]

Supplementary Table 1
the genome coordinates of up-regulated lncRNAs

LncRNA	genome coordinates	Strand
FCGR2A-212	chr1:161487765-161492889	+
ILF3-205	chr19:10798281-10803074	+
IP6K1-206	chr3:49822913-49823627	-
SLC6A6-206	chr3:14444148-14499534	+
PRKACA-202	chr19:14202509-14219202	-
CSNK1G3-204	chr5:122847885-122881650	+
TFDP1-205	chr13:114239058-114288314	+
RPS10-204	chr6:34385231-34393561	-
ZMYM1-202	chr1:35544992-35581455	+
KCNE1-207	chr21:35881754-35884505	-
UXT-204	chrX:47511200-47516898	-
TRAF3IP3-208	chr1:209929481-209935941	+
GFOD1-205	chr6:13469500-13472276	-
GSTO1-206	chr10:106013922-106025948	+
C22orf34-203	chr22:50014119-50051078	-
CTBP2-206	chr10:126691907-126848714	-
LINC00847-202	chr5:180258365-180262678	+
AL627309.1-205	chr1:120725-133723	-
LINC01002-214	chr19:199835-200736	-
CCDC88B-208	chr11:64111011-64117859	+
MEF2C-250	chr5:88067606-88100436	-
AC127502.1-204	chr15:30776747-30799826	+
ADGRE2-211	chr19:14879603-14887653	-
PSME4-208	chr2:54163232-54197915	-
DGAT2-209	chr11:75479844-75485087	+
LAMTOR2-205	chr1:156024876-156028297	+
ALDH1A2-214	chr15:58538382-58623829	-
MYO15B-211	chr17:73586422-73587327	+
HTRA2-206	chr2:74756902-74760374	+
RBM3-207	chrX:48432872-48434807	+
PDE3B-203	chr11:14810485-14840752	+
FAM157C-202	chr16:90224580-90225984	+
FOXO4-204	chrX:70316103-70320622	+
SLED1-201	chr4:185719450-185720200	-
SIRPB2-207	chr20:1459005-1461081	-
FGR-207	chr1:27949895-27961645	-
XYLT1-203	chr16:17448529-17497358	-
DENND3-216	chr8:142165993-142176332	+

CCDC88B-204	chr11:64107695-64124996	+
ANP32A-205	chr15:69105285-69110054	-
PCNX1-212	chr14:71517375-71527459	+
LINC01270-202	chr20:48927511-48928551	+
LINC00963-204	chr9:132264269-132275965	+
LINC01002-220	chr19:198264-200507	-
LINC02193-207	chr16:90239625-90252612	+
GTDC1-214	chr2:144764901-144903472	-
AC104695.2-201	chr2:28648812-28649586	+
GOLGA8B-205	chr15:34837589-34875852	-
MMP25-202	chr16:3097532-3106163	+
USB1-215	chr16:58035320-58048214	+
LINC01270-201	chr20:48909257-48931459	+
NSFL1C-210	chr20:1433765-1454487	-
TLE4-215	chr9:82278365-82323077	+
LINC00957-201	chr7:44078770-44080702	+
VCAN-209	chr5:82831433-82877155	+
GBA-211	chr1:155204807-155211022	-
ALOX5-203	chr10:45938690-45939680	+
LINC00963-211	chr9:132251191-132265709	+
NOL8-212	chr9:95063861-95071232	-
FAM19A2-209	chr12:62464203-62653535	-
VCAN-207	chr5:82837767-82849293	+
MCTP2-202	chr15:95019609-95022812	+
GOLGA3-204	chr12:133404410-133405317	-
MFSD14C-202	chr9:99660348-99711867	-
PNN-206	chr14:39648294-39651954	+
LINC00937-205	chr12:8536701-8549091	-
EYA3-205	chr1:28337307-28362190	-
SLC25A16-202	chr10:70237756-70240521	-
FAM157C-204	chr16:90224340-90244752	+
ARFGAP1-205	chr20:61915644-61921140	+
DAXX-207	chr6:33287310-33290707	-
HDGF-209	chr1:156713633-156721288	-
OGDH-208	chr7:44706220-44714844	+
FAM157B-201	chr9:141107518-141143444	+
SLA-217	chr8:134060824-134087198	-
VSIR-203	chr10:73520239-73521701	-
SYAP1-202	chrX:16737894-16754644	+
ZFAND3-205	chr6:38110773-38120771	+
DPF3-205	chr14:73190340-73209419	-
BCR-205	chr22:23651661-23660221	+

MEF2C-251	chr5:88068099-88100434	-
Z97192.2-201	chr22:49965912-49969074	-
ALOX5-206	chr10:45938869-45939662	+
MIATNB-207	chr22:27113215-27138801	+
PDLIM7-214	chr5:176910876-176912015	-
MKL1-207	chr22:40859232-41032691	-
SLTM-213	chr15:59192616-59225757	-
GRAMD1A-213	chr19:35491276-35500349	+
PPP2R5C-217	chr14:102348542-102378937	+
STX4-211	chr16:31044416-31051450	+
FAM157C-201	chr16:90168679-90204401	+
SEPT14P19-201	chr19:191212-195696	-
E2F2-202	chr1:23842851-23845888	-
NLRP3-207	chr1:247581922-247589094	+
ITSN1-230	chr21:35126488-35144604	+
FLVCR1-AS1-203	chr1:213029946-213031430	-
PTMS-204	chr12:6874682-6880041	+
PHF12-208	chr17:27238151-27239804	-
SYNGAP1-226	chr6:33394593-33399791	+
TNFRSF1A-206	chr12:6439434-6440313	-
AL627309.5-203	chr1:146386-173862	-
TAZ-208	chrX:153641058-153641904	+
DENND3-220	chr8:142183604-142186751	+
DYSF-212	chr2:71797268-71798046	+
CREB5-214	chr7:28725754-28818845	+
ITPA-207	chr20:3202399-3204516	+
ST3GAL2-203	chr16:70432986-70456976	-
E2F3-IT1-201	chr6:20438052-20440409	+
AC240565.1-201	chr11:150836-180404	-
MYH9-211	chr22:36696956-36697760	-
GALNS-207	chr16:88906743-88923250	-
AC068580.1-201	chr11:1784239-1784979	-
UPF1-211	chr19:18959817-18963105	+
LRMDA-210	chr10:77360998-77795894	+
LINC00937-203	chr12:8535440-8538323	-
IQSEC1-202	chr3:12978051-13036626	-
LINC00862-204	chr1:200335719-200369833	-
AL669831.1-201	chr1:661265-714006	-
AL669831.3-214	chr1:655433-659944	-
HERC2P2-205	chr15:23282281-23356187	-
SCFD1-214	chr14:31121544-31164064	+
SNHG9-201	chr16:2014960-2015510	+

SMG1P4-202	chr16:21912873-21922693	-
NADK-210	chr1:1710596-1711896	-
GBA-205	chr1:155207723-155208682	-
GDPD5-210	chr11:75236329-75236909	-
CCND3-210	chr6:41904387-41906004	-
STAT3-214	chr17:40538957-40540439	-
AC104695.3-201	chr2:28619682-28619977	+
TSPAN32-209	chr11:2323270-2337186	+
FAM223A-201	chrX:153799479-153800186	+
AC135050.3-201	chr16:31054471-31061189	+
PSTPIP1-216	chr15:77287703-77317659	+
NAPA-203	chr19:47999030-48018296	-
CD9-209	chr12:6341352-6345428	+
DENND3-209	chr8:142187186-142195322	+
WWP2-217	chr16:69924701-69974524	+
GUSBP11-204	chr22:23980674-24059508	-
AC131009.4-201	chr12:132381045-132382784	+
PAXIP1-206	chr7:154735494-154793810	-
ARHGAP27P1-BPTFP1-KPNA2P3-201	chr17:62745781-62777744	-
ATF6B-212	chr6:32093819-32095995	-
WDR1-218	chr4:10076773-10094643	-
CYBA-202	chr16:88714101-88717462	-
ZNF815P-205	chr7:5862847-5879673	+
DGKD-211	chr2:234356347-234357801	+
AC116353.5-204	chr5:139956533-139973337	-
UPF1-205	chr19:18959891-18963045	+
CSNK1D-225	chr17:80202598-80202989	-
DDX46-208	chr5:134117760-134130658	+
SLA-205	chr8:134085153-134088372	-
AP002761.4-201	chr11:72950020-72953380	+
AC097376.1-201	chr4:140347453-140348449	-
KDM4B-212	chr19:5074102-5079177	+
AP001922.3-201	chr11:75486949-75487974	+
AC132938.6-201	chr17:80420477-80422131	+
DNAJB6-214	chr7:157129698-157164921	+
AC116407.4-201	chr17:30486330-30487390	+
AL442125.1-201	chr13:114181575-114184936	+
HERC2P2-203	chr15:23308629-23311662	-
Z98749.2-205	chr22:38746382-38794865	-
VNN2-210	chr6:133074592-133079012	-
ANAPC5-212	chr12:121766802-121775198	-
LINC00937-207	chr12:8448582-8549399	-

FES-208	chr15:91432780-91434252	+
RABGGTB-205	chr1:76252384-76255681	+
SRGAP2-006	chr1:206626691-206627855	+
MACF1-210	chr1:39927696-39945788	+
UPF1-209	chr19:18959975-18963063	+
ADSS-203	chr1:244574669-244582575	-
PCNX1-206	chr14:71374387-71409657	+
ADCY10P1-201	chr6:41107665-41108570	+
C22orf34-201	chr22:50013290-50018568	-
AP3S2-205	chr15:90380850-90395888	-
ZFAND5-207	chr9:74975354-74979892	-
AC006001.2-202	chr7:66119538-66134285	+
KDM8-203	chr16:27214838-27224963	+
MICAL1-207	chr6:109772932-109773734	-
AIDA-203	chr1:222843532-222885605	-
SULF2-202	chr20:46290178-46292298	-
PRPF4B-208	chr6:4061216-4062303	+
EPG5-203	chr18:43496003-43496770	-
CRLS1-204	chr20:6008917-6015201	+
RNF130-203	chr5:179382480-179383516	-
AC245884.11-201	chr19:54820182-54848439	-
METTL8-211	chr2:172193982-172291312	-
SLC22A4-203	chr5:131631118-131656665	+
AC025280.1-201	chr16:84861869-84862848	+
B4GALT4-210	chr3:118949003-118959502	-
AL158166.1-201	chr10:129732962-129734431	+
AP3S2-215	chr15:90378829-90392239	-
FAM160A2-204	chr11:6232575-6236585	-
DYSF-215	chr2:71803398-71827970	+
AC093159.1-201	chr2:62690262-62691205	+
ARMCX3-204	chrX:100878165-100879522	+
SLC43A3-209	chr11:57182687-57193952	-
NPL-210	chr1:182758908-182785882	+
AP001434.1-201	chr21:39609139-39610123	-
PRDM5-211	chr4:121606074-121631566	-
RNF19A-204	chr8:101312806-101322376	-
SEPT9-243	chr17:75372003-75398171	+
SMG1P1-207	chr16:22448329-22472134	+
GTPBP1-204	chr22:39121181-39122433	+
SLC25A16-201	chr10:70242776-70277975	-
RBM23-225	chr14:23375410-23388358	-
CIZ1-218	chr9:130929038-130953829	-

NADSYN1-223	chr11:71214910-71216920	+
LINC00266-1-201	chr20:62921738-62934912	+
TLR5-204	chr1:223307774-223316588	-
AL627309.5-204	chr1:165889-168767	-
MGAT4B-210	chr5:179229952-179232385	-
ALDH9A1-205	chr1:165637911-165649872	-
ECE1-206	chr1:21599328-21671630	-
MLLT1-203	chr19:6210700-6211487	-
KDM4B-207	chr19:5080699-5110745	+
MIIP-203	chr1:12082279-12092032	+
NRDC-211	chr1:52305664-52344443	-
AC060780.1-203	chr17:41317777-41322458	-
ABTB1-204	chr3:127391857-127393444	+
CNOT3-207	chr19:54653465-54657556	+
NPIPB5-012	chr16:22524885-22530556	+
SEPT2-202	chr2:242263631-242276513	+
LINC02356-202	chr12:111807086-111841114	+
BCL3-205	chr19:45258406-45260425	+
CHSY1-206	chr15:101718807-101728075	-
PABPC1L-214	chr20:43566484-43567958	+
AC093752.1-210	chr4:120415620-120419182	+
PIAS1-208	chr15:68484020-68490941	+
AL078604.2-201	chr6:160007987-160010201	-
PARP8-205	chr5:49963727-50138176	+
CMIP-205	chr16:81678965-81685895	+
AC092910.3-202	chr3:119814117-119818033	+
DYSF-217	chr2:71829814-71840506	+
AC009093.8-202	chr16:29126038-29229181	+
MSRB1-204	chr16:1990979-1991940	-
DNTTIP2-203	chr1:94341222-94345474	-
NECAP2-205	chr1:16767231-16768114	+
ALOX5AP-202	chr13:31316473-31318533	+
GYS1-203	chr19:49490451-49496540	-
DCAF7-204	chr17:61627871-61629916	+
SNHG4-206	chr5:138609820-138614818	+
GLA-203	chrX:100653504-100655841	-
HSPBAP1-206	chr3:122478067-122512507	-
DGKD-209	chr2:234317537-234344570	+
STAG3L5P-202	chr7:99933748-99938951	+
HERC4-212	chr10:69826939-69834614	-
SERF1B-207	chr5:69321414-69328320	+
PHF21A-213	chr11:46141940-46142609	-

EIF4G3-213	chr1:21501607-21503312	-
USP53-208	chr4:120133898-120141366	+
TTC7A-208	chr2:47205257-47233133	+
DNTTIP2-206	chr1:94342315-94344712	-
TDRD9-207	chr14:104406137-104433165	+
KNTC1-207	chr12:123077365-123087191	+
CARD8-205	chr19:48737103-48752871	-
PDXDC2P-NPIPB14P-201	chr16:70026923-70055833	-
Z98749.2-202	chr22:38732342-38740439	-
ARHGEF2-214	chr1:155916646-155972355	-
KIAA0319L-206	chr1:35900905-35909831	-
PKD1P6-205	chr16:15227345-15229010	-
N4BP2L2-206	chr13:33018103-33092057	-
CEACAM1-212	chr19:43013252-43016563	-
LINC01481-201	chr12:70612912-70615642	-
DHRSX-IT1-201	chrX:2252336-2254451	-
COMTD1-205	chr10:76994184-76995094	-
ACAP2-202	chr3:195053811-195076941	-
OXLD1-205	chr17:79632086-79633176	-
NRDC-207	chr1:52260144-52261380	-
GBA2-208	chr9:35744294-35748970	-
LINC02256-201	chr15:32828915-32867844	+
UBE2Q1-203	chr1:154524227-154524851	-
CCDC93-204	chr2:118709065-118753865	-
IFNGR1-204	chr6:137527779-137540586	-
MARC1-206	chr1:220964297-220965006	+
SFXN3-205	chr10:102797709-102800997	+
PPHLN1-215	chr12:42719983-42753460	+
ARMCX6-206	chrX:100870108-100872900	-
DENND2D-206	chr1:111740541-111747157	-
INPP5A-206	chr10:134503896-134540853	+
TNFRSF1B-202	chr1:12251895-12253065	+
PLCG2-210	chr16:81832071-81902838	+
SQSTM1-214	chr5:179238615-179240641	+
CCDC34-203	chr11:27352374-27371971	-
SFXN5-216	chr2:73280458-73298791	-
AC104561.3-201	chr8:23350522-23351711	-
GPR141-205	chr7:37723469-37753112	+
AL137145.1-201	chr10:6392278-6394724	+
TFRC-208	chr3:195801068-195808961	-
NUP50-AS1-202	chr22:45530171-45559540	-
AC093330.2-201	chr18:74714805-74718802	-

AC116353.4-202	chr5:139943475-139953782	-
IGF2BP2-208	chr3:185414203-185542680	-
ANXA11-208	chr10:81932590-81965278	-
ILK-213	chr11:6625026-6625956	+
SRGAP2B-002	chr1:143913615-144094424	+
ETV6-203	chr12:11802878-11979490	+
SH3BP5L-203	chr1:249104645-249108372	-
NDUFA3-211	chr19:54611854-54612236	+
JAK3-202	chr19:17949079-17958841	-
IGF1R-205	chr15:99496024-99500521	+
MYOM1-206	chr18:3067263-3075649	-
MEG8-201	chr14:101415315-101424887	+
AZIN1-209	chr8:103848474-103870272	-
ZRSR2-203	chrX:15808635-15821919	+
INTS8-202	chr8:95876921-95884115	+
AC009090.5-201	chr16:57176564-57178848	+
PQLC1-212	chr18:77663894-77675961	-
CDKN1A-206	chr6:36646548-36648634	+
CTDSP1-209	chr2:219265785-219269496	+
PADI2-203	chr1:17394281-17409921	-
LRRC37A17P-202	chr17:45055847-45126511	+
NOTCH1-202	chr9:139417080-139418350	-
GTF3C5-208	chr9:135929290-135930734	+
CRYBB2P1-202	chr22:25844072-25857645	+
ARRDC1-210	chr9:140505903-140509811	+
UBAC2-205	chr13:99965037-100038688	+
LINC01002-203	chr19:201360-205598	-
BRD2-217	chr6:32938482-32939825	+
FXR2-204	chr17:7494760-7495675	-
SH3PXD2B-204	chr5:171760503-171761239	-
CAB39-205	chr2:231643721-231655693	+

Supplementary Table 2

the genome coordinates of down-regulated lncRNAs

Trans_Name	Locus	Strand
YBX3-210	chr12:10851916-10855074	-
GUCD1-212	chr22:24938798-24940051	-
CPNE1-223	chr20:34246852-34252822	-
NPEPPS-204	chr17:45600311-45669911	+
TMEM50A-205	chr1:25664811-25687756	+
GAS5-209	chr1:173833039-173838020	-
LINC02446-201	chr12:10705962-10710648	+
LINC01506-202	chr9:71158457-71161505	-
DDX11L9-201	chr15:102516761-102519296	-
BID-206	chr22:18220824-18257261	-
CLHC1-210	chr2:55445256-55459399	-
CNIH4-208	chr1:224544623-224558999	+
RAP1A-204	chr1:112170091-112255318	+
LINC00534-202	chr8:91233716-91400216	+
RPL30-204	chr8:99055059-99056360	-
TBC1D20-204	chr20:416927-443187	-
SARS-205	chr1:109756590-109778053	+
GUK1-214	chr1:228328833-228335411	+
CD72-208	chr9:35615940-35618352	-
SLC25A37-210	chr8:23424196-23429103	+
GAB1-206	chr4:144361326-144387328	+
TRNAU1AP-206	chr1:28887145-28904287	+
GOLGA8B-208	chr15:34845108-34875852	-
ANAPC10-207	chr4:145888264-146019365	-
SEC22A-205	chr3:122920774-122962797	+
ADAMTSL4-205	chr1:150521897-150524382	+
PCED1B-AS1-209	chr12:47599681-47610191	-
BX284668.5-202	chr1:17215037-17216137	-
LINC00969-250	chr3:195415238-195423412	+
PUM2-205	chr2:20508175-20550595	-
NDEL1-206	chr17:8339179-8350098	+
EXD2-212	chr14:69658246-69709074	+
ATG7-221	chr3:11314129-11346023	+
NDUFV2-206	chr18:9117524-9119564	+
EHMT1-241	chr9:140514499-140605482	+
LPCAT2-205	chr16:55590835-55616924	+
SERTAD3-205	chr19:40946754-40947292	-
NDUFV2-203	chr18:9119471-9134339	+

H2AFY-203	chr5:134670107-134734912	-
UBB-207	chr17:16284409-16286059	+
RPL39-203	chrX:118920467-118921419	-
